# Supplementary material for: The Microbiological Characteristics and Genomic Surveillance of Carbapenem-Resistant Klebsiella pneumoniae Isolated from Clinical Samples
Source: Microorganisms. 2025 Jul 4;13(7):1577. doi: 10.3390/microorganisms13071577 (PMC12299758; doi:10.3390/microorganisms13071577)
Supplement: Supplementary file 1 [file microorganisms-13-01577-s001.zip › microorganisms-3625976-supplementary.pdf]

Supplementary Materials:

Table S1. Virulence genes associated with carbapenam resistant *Klebsiella pneumoniae*:

VFclass	**Virulence factors**	**Related genes**	**N**
Adherence	Type III fimbriae	*mrkA*	24
		mrkB	24
		mrkC	24
		mrkD	24
		mrkF	24
		mrkH	24
		mrkI	24
		mrkJ	24
	Type I fimbriae	*fimA*	24
		fimB	24
		fimC	24
		fimD	24
		fimE	24
		fimF	24
		fimG	24
		fimH	24
		fimI	24
		fimK	24
	D-alanine-polyphosphoribitol ligase(Listeria)	*dltA*	2
	Streptococcal plasmin receptor/GAPDH (Streptococcus)	*plr/gapA*	2
	Type IV pili(Yersinia)	*pilW*	
Antiphagocytosis	Capsule	*Capsule*	24
Efflux pump	AcrAB	*acrA*	24
		acrB	24
Iron uptake	Aerobactin	*iutA*	24
	Ent siderophore	*entA*	24
		entB	24
		entC	24
		entD	24
		entE	24
		entF	24
		entS	24
		fepA	24
		fepB	24
		fepC	24
		fepD	24
		fepG	24
		fes	24
	Salmochelin	*iroE*	24
		iroN	24
	Yersiniabactin	*fyuA*	19
		irp1	19
		irp2	19
		ybtA	19

		ybtE	19
		ybtP	19
		ybtQ	19
		ybtS	19
		ybtT	19
		ybtU	19
		ybtX	19
	Ferrous iron transport (Shigella)	*sitB*	1
		sitC	19
	Heme biosynthesis (Haemophilus)	*hemL*	1
	Periplasmic binding protein-dependent ABC transport systems (Vibrio)	*vctC*	1
	Iron/manganese transport(Escherichia)	*sitA*	6
		sitD	12
Regulation	RcsAB	*rcsA*	24
		rcsB	24
Secretion system	T6SS-I	*clpV/tssH*	24
		dotU/tssL	24
		hcp/tssD	24
		icmF/tssM	24
		impA/tssA	10
		ompA	22
		sciN/tssJ	24
		tssF	24
		tssG	24
		vasE/tssK	24
		vgrG/tssI	24
		vipA/tssB	24
		vipB/tssC	24
	T6SS-II	*clpV*	24
	T6SS-III	-	17
		-	9
		dotU	24
		icmF	24
		impA	24
		impF	24
		impG	24
		impH	24
		impJ	24
		ompA	24
		sciN	24
		vgrG	24
Serum resistance	LPS rfb locus	*LPS rfb locus*	24
Fimbrial adherence determinants	Ste(Salmonella)	*steB*	10
	Stf(Salmonella)	*stfD*	9
	Stb(Salmonella)	*stbA*	10
		stbB	10
		stbC	10
		stbD	10
		stbE	10
Stress adaptation	Manganese transport system(Neisseria)	*mntB*	1

Acid resistance	Urease(Helicobacter)	*ureB*	1
Anaerobic respiration	Nitrate reductase (Mycobacterium)	*narG*	1
		narH	1
Cell surface components	Trehalose-recycling ABC transporter (Mycobacterium)	*sugC*	1
Enzyme	Streptococcal enolase(Streptococcus)	*eno*	1
Immune evasion	Capsule(Staphylococcus)	*capL*	1
	Capsule(Streptococcus)	*cps4I*	1
	Polysaccharide capsule(Bacillus)	*gtaB*	1
		lytR	1
		manA	1
Iron acquisition	Bacillibactin(Bacillus)	*dhbA*	1
		dhbC	1
Stress adaptation	Catalase (Neisseria)	*katA*	1
Lipid and fatty acid metabolism	Isocitrate lyase (Mycobacterium)	*icl*	1

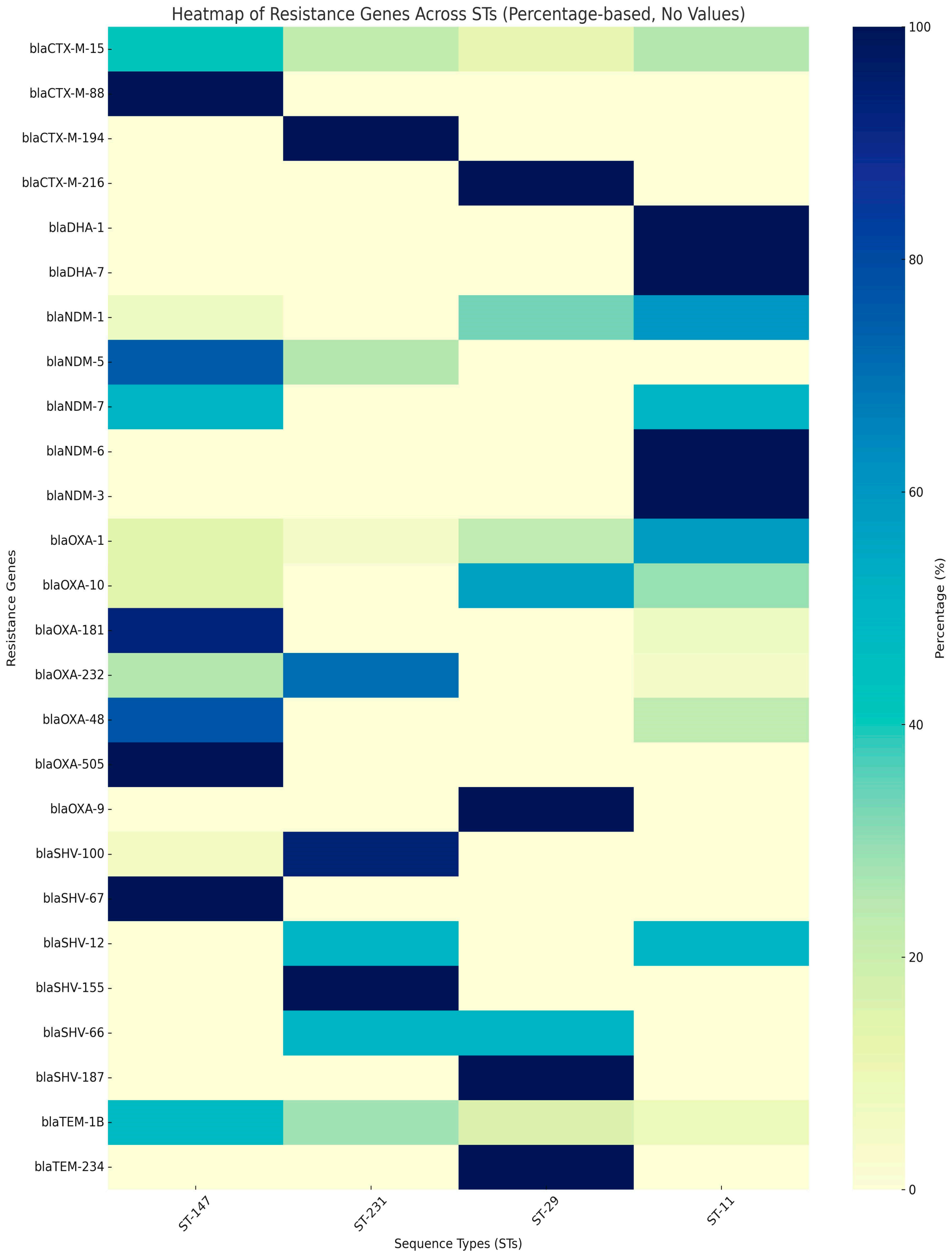

Figure S1. Percent Distribution of antimicrobial resistance genes among different sequence types.
